# Resetting predator baselines in coral reef ecosystems

**DOI:** 10.1038/srep43131

**Published:** 2017-02-21

**Authors:** Darcy Bradley, Eric Conklin, Yannis P. Papastamatiou, Douglas J. McCauley, Kydd Pollock, Amanda Pollock, Bruce E. Kendall, Steven D. Gaines, Jennifer E. Caselle

**Affiliations:** 1 Bren School of Environmental Science and Management, University of California Santa Barbara, Santa Barbara, CA 93106, USA; 2 The Nature Conservancy, Hawai’i, 923 Nu’uanu Avenue, Honolulu, HI 96817, USA; 3 Department of Biological Sciences, Florida International University, North Miami, FL 33181, USA; 4 Department of Ecology, Evolution, and Marine Biology, University of California Santa Barbara, Santa Barbara, CA 93106, USA; 5 U.S. Fish and Wildlife Service, 300 Ala Moana Blvd, Honolulu, HI 96850, USA; 6 Marine Science Institute, University of California Santa Barbara, Santa Barbara, CA 93106, USA

## Abstract

What did coral reef ecosystems look like before human impacts became pervasive? Early efforts to reconstruct baselines resulted in the controversial suggestion that pristine coral reefs have inverted trophic pyramids, with disproportionally large top predator biomass. The validity of the coral reef inverted trophic pyramid has been questioned, but until now, was not resolved empirically. We use data from an eight-year tag-recapture program with spatially explicit, capture-recapture models to re-examine the population size and density of a key top predator at Palmyra atoll, the same location that inspired the idea of inverted trophic biomass pyramids in coral reef ecosystems. Given that animal movement is suspected to have significantly biased early biomass estimates of highly mobile top predators, we focused our reassessment on the most mobile and most abundant predator at Palmyra, the grey reef shark (*Carcharhinus amblyrhynchos*). We estimated a density of 21.3 (95% CI 17.8, 24.7) grey reef sharks/km^2^, which is an order of magnitude lower than the estimates that suggested an inverted trophic pyramid. Our results indicate that the trophic structure of an unexploited reef fish community is not inverted, and that even healthy top predator populations may be considerably smaller, and more precarious, than previously thought.

Wildlife management and conservation is fundamentally concerned with assessing the status of wild populations today and forecasting their trends into the future. Two bounding points of reference can play critical roles for different decisions – historical abundances prior to human impacts and population extinction. On land, human impacts have been ubiquitous and enduring for centuries^1^,^2^,^3^,^4^, and habitats are so altered that recovering species to historical levels is generally unfeasible and rarely even imagined. Instead, terrestrial conservation biologists are disproportionately concerned with the other end of the abundance spectrum – reducing risks of extinction^5^. By contrast, extinctions in the sea are still comparatively rare^6^,^7^, but thousands of species are harvested from wild populations, often at rates that are currently unsustainable^8^,^9^. Setting harvest targets that maintain vibrant populations of targeted species and the broader structure of the ecosystem in which they thrive requires a benchmark understanding of marine ecosystems^10^,^11^,^12^. Reconstructing what a pristine ocean looked like is therefore critical to set appropriate management targets for fished species *and* to recover threatened species^10^,^13^.

Unsurprisingly, a great deal of effort has gone into estimating historical baselines of ocean abundance. Most estimates have come from indirect measures, such as reconstructing fisheries landings trajectories to hindcast pre-exploitation abundances (e.g. refs 12, 14, 15, 16) or using patterns of genetic diversity to infer historical population sizes^17^,^18^. However, humans began aggressively removing species from the ocean well before we began studying them, making it generally impossible to establish an accurate historical baseline of population abundance for most marine species solely using data from a post-industrial fishing world^19^. A third method of estimating historical population baselines hinges upon the fact that – unlike on land – there are still places in the ocean that are relatively pristine. Efforts to reconstruct baseline information about marine populations and ecosystem dynamics have focused on the remote central Pacific Ocean^20^,^21^,^22^,^23^, where the Pacific Remote Islands are protected under U.S. jurisdiction and provide a potential window into the historical ocean. Early studies in one such isolated system, Palmyra atoll, determined that the grey reef shark (*Carcharhinus amblyrhynchos)*, the most abundant predator in terms of biomass^24^, had a density that ranged from ~200 sharks/km^2^ (towed-diver survey^25^ and SCUBA belt transects^24^) to over 1000 sharks/km^2^ (SCUBA belt-transect survey^25^ calculated from shark biomass reported in ref. 23). The latter estimate provided support for the surprising and controversial idea that historical marine food webs may have had an inverted trophic pyramid with larger biomass at the top of the food web than at trophic levels below^21^,^22^,^23^.

If this vision of past ocean ecosystems was correct, and if it applied to other ocean geographies and habitats, the implications for ocean management and the need for ocean restoration would be profound. Not surprisingly, this rather radical scientific revision to our understanding of trophic structure and biomass organization in coral reef ecosystems has also evoked considerable scientific skepticism. Theoretical examinations of biomass distribution and energy flow in ecosystems^26^ questioned the potential validity of the empirical findings. Such conceptual challenges raised the specter of empirical errors, especially with respect to sampling highly mobile but spatially patchy predators with classical approaches^25^,^27^. One path to resolution is to solve the empirical sampling challenges and see if better estimates from pristine habitats in the ocean support a different view of unexploited ocean food webs.

To this end, we set out to re-estimate shark abundance and density at the same unfished coral reef – Palmyra atoll – that inspired the idea of inverted trophic biomass pyramids in coral reef ecosystems. Palmyra atoll is a U.S. National Wildlife Refuge in the central Pacific that was established in 2001 and is one of the few remaining unfished coral reef ecosystems on the planet. Animal movement is suspected to have significantly biased the early biomass estimates at Palmyra, with the reported bias exaggerated for top predators, which tend to be highly mobile^27^. We therefore focused our reassessment on the most mobile and most abundant predator at Palmyra^24^, the grey reef shark, and used survey techniques and analytical methods that can be applied over large spatial areas while accounting explicitly for movement on and off of study areas by target species as well as heterogeneous probability of detection^28^,^29^,^30^. With data from an eight-year, spatially explicit tag-recapture field program, we used spatial capture-recapture (SCR) models to estimate population abundance, density, and individual movement using precise capture-recapture locations and predictions based on standard detection models (Supplementary Methods). We conducted a Bayesian analysis of a SCR model to produce the first spatially and temporally explicit island-wide baseline population abundance and density estimates for a dominant reef shark at an unexploited coral reef. Our refined density estimate allows us to reconstruct top predator biomass and consider the consequences for the trophic biomass structure of an unfished foodweb. Most importantly, we then discuss the management implications of inflated baseline abundance and density estimates for shark populations.

## Results

**Figure 1:**
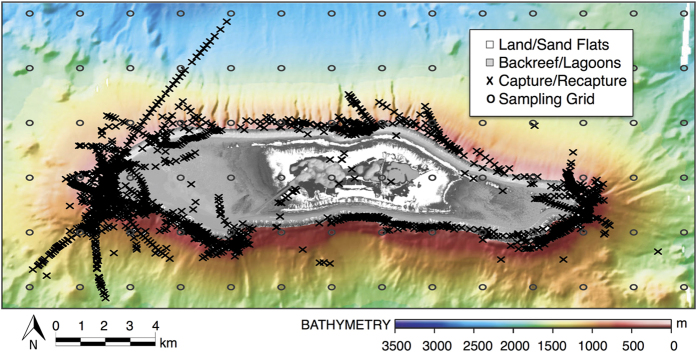
Palmyra Atoll field sampling. A map of Palmyra Atoll, a U.S. Wildlife Refuge with a ban on extractive fishing, showing forereef/offshore, backreef, and lagoon habitats, all of which were sampled during our 8-year capture-recapture study. Capture and recapture locations are shown for all sampling periods (**x**). Circles (**o**) denote the 2 km sampling grid used for the spatial capture-recapture models. Figure was created using R (version 3.1.3 [ www.r-project.org/]). Bathymetry map is from the National Oceanic and Atmospheric Administration’s (NOAA) Coral Reef Ecosystem Division and the Pacific Islands Benthic Habitat Mapping Center data collections 2016 (http://www.soest.hawaii.edu/).

**Figure 2:**
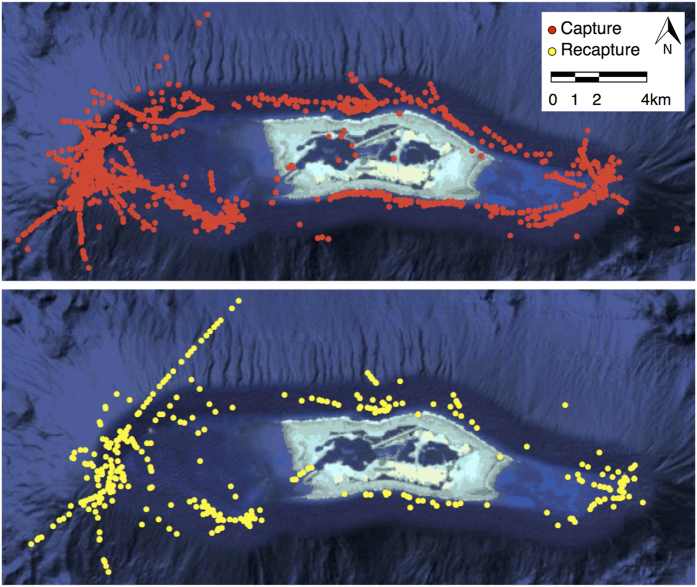
Capture-recapture locations. Capture (N = 1356) and recapture (N = 389) locations for *C. amblyrhynchos* captured over 88 days of handline fishing between 2006–2014. Figure was created using the *ggmap*^61^ function in R (version 3.1.3 [ www.r-project.org/]).

**Figure 3:**
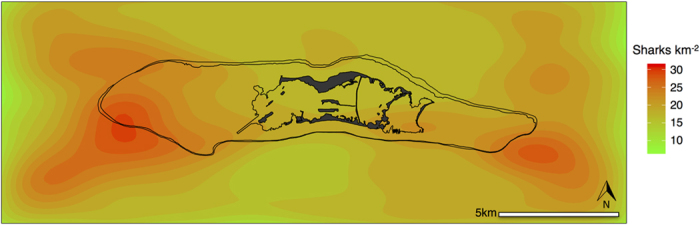
Shark density. Spatially explicit density estimates from capture-recapture data for grey reef shark adults and sub-adults at an unfished coral reef (mean 21.3 sharks/km^2^; 95% CI 17.8–24.7). Density is color-coded (*red* = highest and *green* = lowest). Figure was created using R (version 3.1.3 [ www.r-project.org/]). Habitat information is from NOAA’s NCCOS data collections 2016 (products.coastalscience.noaa.gov).

**Figure 4:**
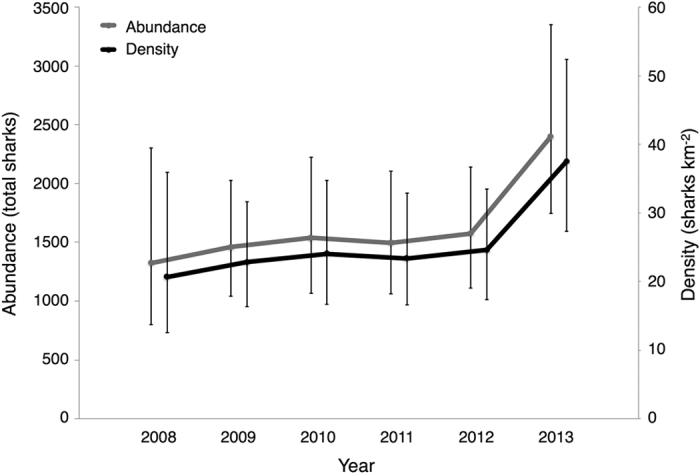
Shark density and abundance through time. Grey reef shark density (black) and abundance (grey) estimates (values reported for 2008–2013 from 2006–2013 capture-recapture data for the western forereef and backreef habitat).

Our tag-recapture field program began in October 2006 and concluded in October 2014. During that time, we captured 1356 individual grey reef sharks (887 female, 469 male, 1 unrecorded) over 88 total days of fishing (Figs 1 and 2; Supplementary Fig. 1). Of these, 389 individuals were recaptured during unique sampling periods (Fig. 2). We examined various parameterizations of our SCR model that included effects of distance from activity center, fishing effort, sex, and size of each individual shark, all of which are known to affect capture probability (e.g. Espinoza *et al*.^31^; Heupel & Simpfendorfer^32^). Given that none of the estimated Bayesian credible intervals (CIs) in the model that included all covariates overlapped zero, we report results from the full model only. Grey reef shark adult and sub-adult density was 21.3 sharks/km^2^ (95% CI 17.8, 24.7), and total population abundance was 8344 individuals (95% CI 6977–9698) (Fig. 3; Supplementary Table 1). The unfished shark population was also stable through time; there were no significant differences in grey reef shark abundance or density through the study period (Fig. 4).

Shark movement was assessed using both the SCR model and multi-year passive acoustic monitoring data available for a subset of the population that was acoustically tagged in 2010–2012 and tracked through August 2015 (N = 37 sharks). Mean activity space estimates from the SCR model and the mean bivariate normal kernel utilization distribution (KUD) estimated from acoustic monitoring were nearly identical at the 99% level (25.2–28.4 km^2^ (95% CI 20.5–31.0 km^2^) and 28.8 km^2^ (95% CI 19.8–37.8 km^2^), respectively). However, there was a significant amount of individual heterogeneity in 99% KUDs estimated from the acoustic data (minimum <1 km^2^, maximum 117.4 km^2^) (Supplementary Table 2). Female sharks also had smaller activity spaces than males (25.2 and 28.4 km^2^ maximum 99% activity space KUD, female and male respectively), indicated by a sharper decline in the detection function at increasing distances from activity centers (Supplementary Table 1). Gelman-Rubin convergence diagnostics reported 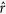 values < 1.1 for all *σ, λ*, and *β* parameters (Supplementary Table 1).

## Discussion

We estimate that grey reef shark density at a near-pristine coral reef is 21.3 sharks/km^2^, which translates to a total population size of 8344 sharks (Supplementary Table 1). These results suggest that unexploited predator density and abundance are substantially lower than was previously reported from the same location (i.e. ~200–1000 sharks/km^2 ^23,24,25). The latter estimate led to the controversial suggestion that pristine coral reefs have inverted trophic pyramids, with disproportionally large top predator biomass. If all species in the system were similarly over-counted, then our finding would not challenge the idea of the inverted trophic biomass pyramid in pristine coral reef ecosystems, because all trophic levels would scale equally. However, it is unlikely that all trophic groups were over-counted equivalently. Ward-Paige *et al*.^27^ used simulation models to demonstrate a direct correlation between animal mobility and over-counting bias in non-instantaneous diver-based underwater surveys: animals that moved faster were over-counted, while slow-moving, sedentary animals were more likely to be accurately assessed.

**Figure 5:**
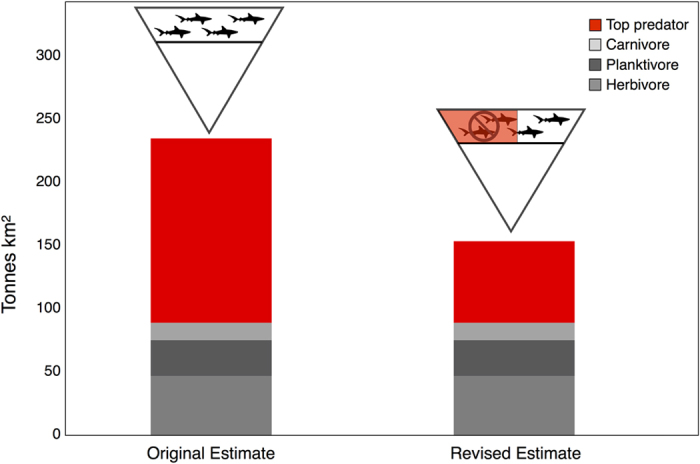
The not-inverted trophic biomass pyramid. The consequences of a 56% reduction to total top predator biomass (area shown in red) based on our density estimates as compared to the numbers used to motivate the claim of an inverted biomass pyramid (original estimate modified from data presented in Sandin *et al*.^23^).

For this reason, we assessed the validity of the inverted trophic pyramid by focusing on the grey reef shark – the species with the highest reported biomass that also happens to be the most mobile predator at Palmyra. Through this lens, our results provide clear evidence that the concept of an inverted trophic biomass pyramid inaccurately represents the overall biomass structure of coral reef ecosystems as a result of overestimated top predator densities likely due to noted sampling biases^25^,^26^,^27^. If all top predators (i.e. sharks) at Palmyra were similarly overestimated by traditional underwater diver surveys due to the mobility bias, our estimate would reduce total top predator biomass by nearly 56% compared to the numbers used as evidence of an inverted biomass pyramid at Palmyra^23^. This substantial biomass reduction reshapes the previously proposed trophic structure into a top-heavy, but not inverted pyramid (Fig. 5), and aligns the biomass profile with known size-based constraints on trophic pyramids^26^. Although it was recently shown that coral reef systems can support inverted trophic biomass pyramids over extremely limited spatial and temporal scales (a single pass during a spawning aggregation)^33^, our results indicate that the inverted pyramid does not accurately describe the entire coral reef ecosystem, as was previously suggested. Our findings are consistent with predator-heavy biomass structures reported for well-protected Mediterranean reefs^34^,^35^ and kelp forest ecosystems^36^, and can likely be explained by energetic subsidies as mobile consumers move across multiple habitats. Palmyra’s top predators are known to consume pelagic as well as nearshore resources, thereby creating important linkages across habitats and expanding the resource base of the nearshore coral reef ecosystem^37^. Ultimately, predator mobility biases both the numerator and the denominator in density estimates. Movement inflates the number of sharks counted by traditional survey methods (*sensu* Ward-Paige *et al*.^27^), while at the same time predator mobility obfuscates the spatial scale of the coral reef foodweb as mobile consumers move between and use resources from multiple habitats (*sensu* Trebilco *et al*.^36^). By accounting for mobility directly and estimating density over space occupied by individuals (via 99% activity space KUDs), our reassessment of shark density and abundance effectively corrects both forms of bias. For species with such complicated behaviors as grey reef sharks, an accurate representation of the coral reef ecosystem and its spatial variability is required to set appropriate management and conservation targets.

We also found no significant trend in grey reef shark population density or abundance through time (Fig. 4), indicating that the population is temporally stable. Grey reef sharks have a reported intrinsic rebound potential of 0.05 yr^−1^ (r_2M_), which equates to a doubling time of 12.8 years^38^. The observed population stability may therefore indicate true population recovery from potential fishing pressure prior to the establishment of the marine refuge in 2001, validating the use of our population estimate as representative of a historical baseline.

An additional important observation to emerge from our spatially extensive sampling design was the discovery of density hotspots on the eastern and western forereefs, with shark densities up to an order of magnitude lower on the north and south forereefs, on the backreefs, and within the lagoons (Fig. 3). This spatial organization would likely bias spatially limited population surveys or surveys conducted with non-random sampling, further highlighting the necessity of comprehensive spatial sampling to establish historical baselines accurately. Interestingly, while shark density is variable across space, grey reef sharks displayed strong residency, consistent with previous findings for relatively isolated populations^39^,^40^,^41^. Activity space estimates from our spatial capture-recapture model were similar to mean 99% KUD estimates from grey reef shark passive acoustic monitoring (25.2–28.4 km^2^ and 28.8 km^2^, respectively; Supplementary Table 2). However, 95% utilization distributions estimated using a Brownian bridge model were substantially smaller at 4.4 ± 1.3 km^2^ and revealed that sharks used core areas that were highly stable over several years (Papastamatiou, unpublished data). We also found that male sharks had slightly larger activity spaces than females (Supplementary Table 1), similarly consistent with published results^31^,^32^; however, all of our acoustically monitored sharks made occasional excursions all or part of the way around Palmyra’s forereefs and/or into backreef and lagoon habitat (J. Caselle & Y. Papastamatiou, unpublished data). A similar observation was made by Heupel and Simpfendorfer^42^, who showed that grey reef sharks maintained discrete home ranges, had consistency in space use through time and a high degree of overlap between 50% and 95% KUDs, but also undertook larger scale movements occasionally. The drivers of these infrequent excursions are currently unknown and merit further investigation, but island-wide movement has important implications for the spatial management of reef sharks and could explain why small marine reserves may be insufficient to maintain and recover reef shark populations^31^,^43^,^44^.

From a conservation perspective, our lower than expected reef shark density and abundance estimates are both troubling and encouraging. If healthy coral reef ecosystems tend to support far fewer sharks than previously thought, then recovering reef shark populations may not be an insurmountable goal. Decline rates for reef shark species (e.g. over 90% reported in Graham *et al*.^45^ and Robbins *et al*.^43^) may have been greatly overestimated for some species of reef shark, if they were based on equally overstated baseline population numbers. This could be good news for sharks and shark conservation. On the other hand, if stable shark populations are indeed smaller than expected as our study has shown, they are consequently more precarious when faced with a given level of harvest. Even with our low intensity, single hook, hand-line fishing, we estimate that we interacted with 16–22% of all grey reef sharks at Palmyra; a short visit by a commercial longline vessel could reset population trajectories for this species in a matter of days. In fact, it has long been reported that fisheries are able to expediently devastate reef shark populations^45^, and over the last decade global shark landings have declined due to population reductions caused by overfishing and poor management^46^,^47^. These fishing-induced population declines may be the result of inflated harvest quotas and insufficient protection status resulting from an overestimated baseline, as has been previously speculated^27^. It is not uncommon for findings from fisheries independent underwater visual census to be translated into management targets for shark populations (e.g. Robbins *et al*.^43^), which could be devastating for these species. To be broadly applicable, the shark population information reported here will need to be contextualized to the broader biotic and abiotic features of Palmyra; however, our finding that an unexploited location with high quality habitat has surprisingly low, but stable, shark abundance implies that population size limitations resulting from density dependence may be as important in explaining shark vulnerability to overexploitation as the slow life history characteristics that are primarily cited.

Marine wildlife management has an opportunity unparalleled in terrestrial systems to reconstruct historical baselines for many species using the few remaining large unexploited marine ecosystems. However, monitoring mobile species in complex ecosystems presents a suite of technical challenges that demand the application of appropriately sophisticated analytics. By employing an assessment method that directly addresses the potential biases associated with counting mobile species, we find that the conclusions that were drawn from methodologically and analytically simplistic methods can be strikingly at odds with reality. Ultimately, overestimates of shark density and biomass represent a double-edged sword that can weaken shark conservation. Our far lower estimates of shark abundance and density suggest that shark recovery targets should be adjusted: present targets are likely unrealistic and therefore impossible to achieve. At the same time, harvest quotas should be similarly downgraded to prevent continued overexploitation of these important predators.

## Methods

### Ethics Statement

This project was certified and all sampling protocols were approved by the Institutional Animal Care and Use Committee (IACUC), University of California, Santa Barbara, protocol no. 856 (date of IACUC approval: 5/31/2012) and under U.S. Fish and Wildlife Service (USFWS) special use permits (permit numbers #12533-14011, #12533-13011, #12533-12011, #12533-11007, #12533-10011, #12533-09010, #12533-08011, and #12533-07006). All methods were carried out in accordance with relevant IACUC and USFWS guidelines and regulations.

### Study Site

Palmyra atoll is a U.S. incorporated territory that was established as a Fish and Wildlife Refuge in 2001 and is currently managed by The Nature Conservancy and the U.S. Fish and Wildlife Service. Palmyra is located in the central Pacific (5°54′N; 162°05′W); the associated marine refuge and marine national monument, within which commercial fishing is banned, extends out 50 nautical miles. Shark fishing within the refuge is scientific and non-extractive.

### Field sampling

Minimally invasive tags with unique number IDs were applied to individual grey reef sharks during 12 separate sampling occasions in the spring, summer, and/or fall from October 2006 through October 2014 (Fig. 2; Supplementary Fig. 1). Between 2006 and 2012, tag application was primarily via use of roto tags (also called fin tags), which are applied with an applicator through a hole punched in the leading edge of the first dorsal fin. Starting in 2010, 15 cm stainless steel head dart tags (Hallprint Co.) were introduced and applied using stainless steel tag applicators, with the head of the tag implanted in the dorsal musculature near the base of the first dorsal fin. Tag loss has been reported for dart tags used on reef sharks^48^, which can significantly bias capture-recapture estimates. To determine rate of tag loss, we double-tagged 79 individuals (from all sampled habitats: forereef, backreef, lagoons), applying a uniquely numbered dart tag to each side of the animal near the first dorsal fin. Of these double-tagged individuals, 18 animals were recaptured at least once in a subsequent sampling period (a minimum of 70 days and a maximum of 495 days after initial tagging). All recaptured individuals that were initially double-tagged had *both* tags intact and in good condition upon recapture, indicating that dart tags had minimal tag loss during the study period.

Fishing effort (days spent scientific fishing) varied in intensity at each sampling occasion (Supplementary Fig. 1). Sequential sampling occasions were a minimum of 58 days apart to decrease the likelihood of behavioral effects (e.g. attraction, habituation, aversion to fishing activity). Sampling was unstructured, and fishing locations were chosen opportunistically to cover Palmyra’s forereef habitat and select channel, lagoon, and backreef habitats (Fig. 1). When fishing in forereef and offshore habitats, the boat was allowed to drift with currents (note drift patterns in Fig. 1). Chum (tuna, wahoo, and/or mackerel) was used to attract sharks to the boat, where they were caught using hand lines baited with a single barbless circle hook and restrained at the side of the boat. For each individual, we recorded length (precaudal, fork, and total), sex, and capture/recapture location (latitude and longitude). Given the highly variable oceanographic conditions at individual locations around the atoll, we were unable to accurately estimate the area of attraction for the bait.

### Spatial Capture-Recapture Models

We carried out a Bayesian analysis of our spatial capture-recapture (SCR) model to relate the known observation process (capture-recapture encounter data) to the unknown ecological process (population size, population density, movement)^49^ and allow for temporary emigration^50^. SCR models differ from traditional capture-recapture models in that they introduce a random effect, which corresponds to the location of an individual’s center of activity during the sampling occasion. Individuals that reside primarily in non-sampled areas will therefore have a low probability of capture, while individuals with activity centers close to sampled areas will have a higher probability of capture; the distance between sampled areas (a grid cell center) and an individual’s activity center ultimately determines the probability of capture. Importantly, SCR models make the assumption that an individual is more likely to be captured in areas closest to its activity center, which is assumed to be fixed through the study period (for a full list of SCR model assumptions see Supplementary Methods).

Given our spatially opportunistic survey method and lack of fixed capture/recapture locations, we estimated a SCR model with methods from Thompson *et al*.^51^ and Russell *et al*.^52^. In their modification to traditional SCR models – which use traps set at fixed locations – they impose a spatial sampling grid on the survey area^51^, and grid cells are used as conceptual traps with capture locations assigned to the center point of the grid cell in which the individual was captured^52^. Grid cell size was selected to allow adequate modeling of spatial heterogeneity due to habitat features and the location of individuals within those habitats^51^. Although grey reef sharks have a relatively high movement capacity, Palmyra has highly variable habitat (Fig. 1). Therefore, we used a grid that encompassed our study area with 84, 2 km × 2 km grid-cells (Fig. 1). If no research fishing occurred in a grid cell during a sampling occasion, we imposed the constraint that the probability of encountering an individual was necessarily 0. We also transformed capture/recapture data into binary encounters for each individual during each sampling occasion to minimize the effects of spatial autocorrelation between grid cells given our relatively small grid cell size. Encounter histories take the form *y*_*ijk*_ for an individual *i*, grid-cell *j*, and sampling occasion *k*, where *y*_*ijk*_ = 1 if individual *i* was encountered in grid-cell *j* during sampling occasion *k*, otherwise *y*_*ijk*_ = 0. Encounter probabilities are a function of distance and they are grid-cell specific^53^ where


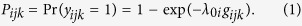



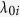
 is the expected number of captures for an individual.

We used a Gaussian hazard model that assumes a circular bivariate normal activity space to model the effect of distance on detection probability as 
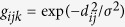
28. The distance between an individual’s activity center and grid-cell center *d* is Euclidean, and *σ* is a scaling parameter^53^. An advantage of the bivariate normal encounter model is that it allows direct estimation of activity space as a function of *σ*^49^. We included four covariates we expected would influence baseline encounter probability: distance from activity center, effort (fishing days), sex, and size (fork length). As sampling intensity varied across sampling occasions, we included fishing effort (days) as a covariate. Sex and size were also included, because they are known to affect activity space in grey reef sharks^31^,^32^ and will therefore impact capture probability. Handline fishing for sharks (the method used in the present study) also tends to result in a female biased sex ratio for captured individuals (Bradley, unpublished data), indicating that capture probability is likely not equivalent in male and female sharks. We therefore ran several parameterizations of the SCR model, introducing each covariate as a linear effect on the linear predictor for detection probability:


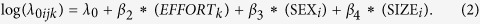


*λ*_0*ijk*_ is then the expected number of captures for an individual *i* in a grid cell *j* given the individual’s activity center at each sampling occasion *k*. All sampled grid-cells have a constant baseline encounter rate *λ*_0_ for the average fishing effort. Both sex and size are unknown for unobserved individuals and were modeled as latent variables with uninformative priors (for model code see Supplementary Methods). We define sex_female_ = 0 and sex_male_ = 1 and report *ψ*_*sex*_ as an estimate of the proportion of the population that is male. To estimate population abundance, we defined a state space S that encompassed our sampling area with individual potential activity centers *s*_*i*_ assigned a prior uniform distribution *s*_*i*_ ~ Uniform(S)^28^,^29^,^53^.

Within the SCR modeling framework, the activity centers of all individuals are estimated as the realization of a point process, and the number of activity centers in a given region represents the population size of that region. We estimated the density of sharks by dividing the total abundance of sharks in the sampled region by the area occupied by those individuals, where area occupied is a function of the activity space of individuals in the population. Changes in population abundance and density were also assessed through time using a subset of data from a region of the island (the western forereef) that was consistently sampled across all sampling occasions (Supplementary Fig. 3). Population time series data were analyzed for the first 9 sampling occasions (2006–2013), because sampling effort was comparable during these occasions (Supplementary Fig. 1).

We constructed the SCR model using data augmentation^54^ with Markov chain Monte Carlo (MCMC) sampling in the *rjags*^55^ package in R^56^. We ran the MCMC algorithm 12,000 times with three chains and discarded the first 2,000 runs as burn-in. Model convergence was assessed using the Gelman-Rubin 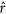 statistic where values < 1.1 indicate convergence^57^. We report only the full model, but results from all model parameterization are reported in Supplementary Table 1.

### Passive Acoustic Telemetry

SCR models make the assumption that activity centers are stationary through the study period. To test the validity of this assumption, and to ground truth SCR model activity space estimates, we examined residency patterns by estimating activity space independent of capture-recapture effort using long-term passive acoustic monitoring data available for a subset of the population. In 2010–2012, 45 grey reef sharks were surgically implanted with acoustic transmitters (69 kHz, V16, Vemco Ltd., Nova Scotia, Canada; for surgical implantation methods see Papastamatiou *et al*.^58^). Passive acoustic telemetry was used to monitor the movement of grey reef sharks via an array of over 70 individual underwater acoustic receivers (VR2W, Vemco) that cover all of Palmyra’s unique forereef and backreef habitats (Supplementary Fig. 2). We calculated 99% bivariate normal kernel utilization distributions (KUDs)^59^ for each acoustically tagged individual with >100 detections and a minimum of 10 months of location data (N = 37) in the package *adehabitatHR*^60^ in R^56^.

## Additional Information

**How to cite this article:** Bradley, D. *et al*. Resetting predator baselines in coral reef ecosystems. *Sci. Rep.* **7**, 43131; doi: 10.1038/srep43131 (2017).

**Publisher's note:** Springer Nature remains neutral with regard to jurisdictional claims in published maps and institutional affiliations.

## Supplementary Material

Supplementary Information
