# Supplementary material for: Resetting predator baselines in coral reef ecosystems
Source: Sci Rep. 2017 Feb 21;7:43131. doi: 10.1038/srep43131 (PMC5318939; doi:10.1038/srep43131)

**Supplementary Information**

**Title:** Resetting predator baselines in coral reef ecosystems

Darcy Bradley, Eric Conklin, Yannis P. Papastamatiou, Douglas J. McCauley, Kydd Pollock,

Amanda Pollock, Bruce E. Kendall, Steven D. Gaines, Jennifer E. Caselle

**Supplementary Methods**

**SCR Model Assumptions.** Spatial capture-recapture (SCR) models – like all capture-recapture models – make a number of assumptions. The first of these is the operational assumption that no tags are lost and all tags are correctly identified (see above). The next set of assumptions relate to statistical aspects of the probability model and the model parameters, and include: (*a*) demographic closure, (*b*) no permanent immigration/emigration (but temporary emigration is allowed), (*c*) activity centers that are randomly distributed and stationary during the sampling period, (*d*) encounters among individuals and for any given individual across the sampling space are independent, (*e*) detection is a declining function of distance from an individual's activity center[1].

*Assumptions (a) & (b).* The assumptions of demographic and geographic closure (i.e. no births, deaths, immigration, or emigration) are routinely violated and our study is no exception. However, provided demographic and geographic changes to the population occur randomly, we can expect a loss of precision, but estimates should remain unbiased[2]. Given the slow life history of the grey reef shark – including late maturity, low fecundity, and an average lifespan of at least 25 years (significantly longer than the study period)[3,4] – we would expect limited loss of precision in parameter estimates due to violations of the demographic closure assumption over the course of our study. Regarding geographic closure, five individual shark tags were recovered post fishing-mortality on Teraina and Tabuaeran Islands (230km and 380km away from Palmyra atoll) and these individuals were removed from the analysis. It is possible that other tagged individuals emigrated from Palmyra; however, we have reason to believe that permanent emigration is limited and sufficiently random to prevent bias in parameter estimates as all

acoustically tagged individuals were detected on the Palmyra array from initial tagging (2011/2012) through 2015.

*Assumptions (c) & (d).* Although SCR models are reasonably robust to violations of assumptions (*a*) and (*b*)[1], we are confident that Assumptions *(c)* and *(d)* are reasonable assumptions for our sampling population. The acoustic telemetry analysis revealed relatively small activity spaces for individual sharks (mean 28.8 ± 4.5 $km^2$, 99% kernel utilization distribution). Furthermore, 95% utilization distributions estimated using a Brownian bridge model were substantially smaller at 4.4 ± 1.3 $km^2$ and revealed that sharks used core areas that were highly stable over several years (Papastamatiou, unpublished data). Taken together, this indicates that activity centers were relatively stationary during the study period. Additionally, we transformed encounter histories into binary encounters for each individual to ensure independence in encounter probability for a given individual during a sampling occasion. Processing time (from capture to release), for each tagged individual was <4 minutes on average, indicating minimal effect of capture of one individual on the probability of capturing other individuals.

*Assumption (e).* Passive acoustic telemetry data was used to ground truth SCR model activity space estimates; no significant differences were found between 99% activity space utilization distributions estimated from the SCR models or the passive acoustic telemetry data.

**SCR Model Code**

```
model {
  sigma[1] ~ dunif(0, 40) # sigma female
```

```
sigma[2] ~ dunif(0, 40) # sigma male
lambda0 ~ dnorm(0,.1)
beta2 ~ dnorm(0,.1)   ## effort
beta3 ~ dnorm(0,.1)   ## sex
beta4 ~ dnorm(0,.1)   ## size

psi~dunif(0,1)
psi.sex ~dunif(0,1)

for(i in 1:M){      # individual level variables; M is the data augmentation parameter
z[i] ~ dbern(psi) # individuals in the population
sex[i] ~ dbern(psi.sex) # prior for unobserved individuals
sex2[i] <- sex[i]+1
size[i] ~ dnorm(-1,1) # prior for unobserved individuals
s[i,1]~dunif(xlim[1],xlim[2])
s[i,2]~dunif(ylim[1],ylim[2])

for(j in 1:J){ # distance function
d2[i,j]<- pow(s[i,1]-x[j,1],2) + pow(s[i,2]-x[j,2],2)

for(k in 1:K){ # the likelihood
logit(p0[i,j,k])<- ifelse(samplingGrid[k,j]==0,0,
                   lambda0+beta1*sex[i]+beta2*effort[k]+ beta3*size[i])
```

```
p[i,j,k]<- z[i]*p0[i,j,k]*exp(-d2[i,j]/(2*sigma[sex2[i]]*sigma[sex2[i]]))
y[i,j,k] ~ dbin(p[i,j,k],K)
} } }
N<-sum(z[])
}
```

## References


1. Royle, J. A., Chandler, R. B., Sollmann, R. & Gardner, B. *Spatial Capture-recapture* (Academic Press, Oxford, United Kingdom, 2014).

2. Kendall, W. L. Robustness of closed capture-recapture methods to violations of the closure assumption. *Ecology* **80,** 2517–2525 (1999).

3. Wetherbee, B. M., Crow, G. L. & Lowe, C. G. Distribution, reproduction and diet of the gray reef shark *Carcharhinus amblyrhynchos* in Hawaii. *Mar. Ecol. Prog. Ser.* **151,** 181–189 (1997).

4. Robbins, W. D. Abundance, demography and population structure of the grey reef shark (*Carcharhinus amblyrhynchos*) and the white tip reef shark (*Triaenodon obesus*) (Fam. Charcharhinidae). PhD thesis, James Cook University. (2006).

5. R Development Core Team. *R A Lang. Environ. Stat. Comput.* (2014).

**Supplementary Table 1. Key parameter estimates from spatial capture-recapture models of grey reef shark abundance and density at an unfished coral reef.**

| Model | Density (sharks/km$^2$) | Abundance (total sharks) | Female $\sigma$[*] | Male $\sigma$ | Both $\sigma$ | $\beta_{effort}$[†] | $\beta_{sex}$[†] | $\beta_{size}$[†] | $\psi_{sex}$[‡] | $\hat{r}$[§] |
|---|---|---|---|---|---|---|---|---|---|---|
| ***Distance + effort + sex + size***[a] | | | | | | | | | | |
| **Mean (SD)** | **21.3 (1.9)** | **8344 (738)** | **2.71 (0.25)** | **2.98 (0.13)** | **0** | **0.16 (0.005)** | **0.80 (0.26)** | **0.15 (0.06)** | **0.44 (0.04)** | **1.08** |
| **95% CI**[b] | **17.8, 24.7** | **6977, 9698** | **2.26, 3.24** | **2.73, 3.25** | **0** | **0.15, 0.17** | **0.29, 1.27** | **0.04, 0.27** | **0.35, 0.53** | **--** |
| *Distance + effort + sex* | | | | | | | | | | |
| Mean (SD) | 21.6 (2.0) | 8485 (768) | 2.7 (0.26) | 2.98 (0.13) | 0 | 0.16 (0.005) | 0.93 (0.22) | 0 | 0.41 (0.04) | 1.09 |
| 95% CI | 17.9, 25.3 | 7032, 9915 | 2.15, 3.19 | 2.74, 3.25 | 0 | 0.15, 0.17 | 0.41, 1.34 | 0 | 0.34, 0.48 | -- |
| *Distance + effort + size* | | | | | | | | | | |
| Mean (SD) | 17.6 (1.3) | 6905 (496) | 0 | 0 | 2.93 (0.12) | 0.16 (0.005) | 0 | 0.26 (0.06) | 0 | 1.01 |
| 95% CI | 15.4, 20.2 | 6024, 7909 | 0 | 0 | 2.71, 3.17 | 0.15, 0.17 | 0 | 0.15, 0.39 | 0 | -- |
| *Distance + sex + size* | | | | | | | | | | |
| Mean (SD) | 21.0 (2.0) | 8214 (782) | 2.64 (0.25) | 2.98 (0.13) | 0 | 0 | 0.66 (0.20) | 0.15 (0.06) | 0.46 (0.04) | 1.07 |
| 95% CI | 17.8, 24.6 | 6662, 9452 | 2.18, 3.16 | 2.75, 3.25 | 0 | 0 | 0.26, 1.04 | 0.04, 0.27 | 0.39, 0.55 | -- |
| *Distance + effort* | | | | | | | | | | |
| Mean (SD) | 17.0 (1.0) | 6671 (399) | 0 | 0 | 2.92 (0.12) | 0.16 (0.005) | 0 | 0 | 0 | 1.01 |
| 95% CI | 15.1, 18.9 | 5906, 7411 | 0 | 0 | 2.71, 3.16 | 0.15, 0.17 | 0 | 0 | 0 | -- |
| *Distance + sex* | | | | | | | | | | |
| Mean (SD) | 21.0 (1.9) | 8244 (729) | 2.70 (0.28) | 2.97 (0.13) | 0 | 0 | 0.89 (0.26) | 0 | 0.42 (0.04) | 1.09 |
| 95% CI | 17.8, 24.6 | 6963, 9633 | 2.20, 3.24 | 2.72, 3.24 | 0 | 0 | 0.38, 1.40 | 0 | 0.33, 0.50 | -- |
| *Distance + size* | | | | | | | | | | |
| Mean (SD) | 17.9 (1.3) | 7028 (523) | 0 | 0 | 2.92 (0.12) | 0 | 0 | 0.27 (0.06) | 0 | 1.01 |
| 95% CI | 15.6, 20.9 | 6102, 8193 | 0 | 0 | 2.68, 3.15 | 0 | 0 | 0.15, 0.39 | 0 | -- |
| *Distance* | | | | | | | | | | |
| Mean (SD) | 17.0 (1.2) | 6669 (467) | 0 | 0 | 2.90 (0.12) | 0 | 0 | 0 | 0 | 1.01 |
| 95% CI | 14.8, 19.4 | 5796, 7615 | 0 | 0 | 2.68, 3.14 | 0 | 0 | 0 | 0 | -- |

[*] $\sigma$ is a scaling factor on the effect of distance in the detection function ($\sigma$ is estimated separately for male and female sharks in models that contain a covariate for sex).

[†] $\beta$ parameters denote the effect of survey effort (days), shark sex, and shark size (fork length, cm) on the probability of detection.

[‡] $\psi_{sex}$ is the probability a grey reef shark in the population is male.

[§] Gelman-Rubin $\hat{r}$ statistics (values <1.1 indicate model convergence).

[a] Full model reported in the text is shown in bold.

[b] Lower and upper 95% Bayesian credible intervals (95% CI).

**Supplementary Table 2. 99% bivariate normal kernel utilization distributions (KUDs) estimated for grey reef sharks at Palmyra atoll from multi-year passive acoustic monitoring data to validate SCR model assumptions (n=37).**

| Transmitter ID* | 99% KUD ($km^2$) | Sex | Size (FL cm) | Capture date | Detection time (days) |
|---|---|---|---|---|---|
| A69.1303.61342 | 7.75 | M | 108 | 9/11/10 | 1541 |
| A69.1303.61338 | 5.23 | F | 124 | 9/13/10 | 1541 |
| A69.1303.61339 | 8.18 | F | 115 | 9/13/10 | 1545 |
| A69.1303.61343 | 58.78 | F | 103 | 9/13/10 | 1542 |
| A69.1303.61340 | 12.91 | F | 91 | 9/15/10 | 1544 |
| A69.1303.61341 | 6.2 | F | 107 | 9/15/10 | 991 |
| A69.1303.61334 | 34.46 | F | 127 | 9/16/10 | 499 |
| A69.1303.61335 | 14.01 | M | 125 | 9/16/10 | 1543 |
| A69.1303.61337 | 38.09 | F | 142 | 9/16/10 | 1318 |
| A69.1303.61336 | 6.33 | F | --† | 9/17/10 | 1506 |
| A69.1303.43870 | 9.65 | F | 139 | 3/30/11 | 690 |
| A69.1303.43876 | 1.88 | F | 117 | 3/30/11 | 1120 |
| A69.9001.30702 | 6.61 | F | 100 | 8/3/11 | 864 |
| A69.9001.30705 | 3.06 | M | 106 | 8/3/11 | 1339 |
| A69.9001.30707 | 15.79 | F | 130 | 8/3/11 | 1313 |
| A69.9001.30708 | 0.11 | F | 129 | 8/3/11 | 1327 |
| A69.9001.30711 | 24.5 | F | 120 | 8/3/11 | 1331 |
| A69.9001.30713 | 0.02 | F | 100 | 8/3/11 | 761 |
| A69.9001.30714 | 8.21 | F | 121 | 8/3/11 | 1315 |
| A69.9002.15320 | 58.89 | F | 86 | 8/3/11 | 1309 |
| A69.9002.15322 | 15.39 | F | 123 | 8/3/11 | 1279 |
| A69.9002.15324 | 35.17 | M | 104 | 8/3/11 | 1304 |
| A69.9002.15326 | 29.02 | F | 105 | 8/3/11 | 1282 |
| A69.9002.15328 | 8.76 | F | 137 | 8/3/11 | 452 |
| A69.9001.30703 | 70.34 | M | 109 | 8/5/11 | 1317 |
| A69.9001.30706 | 49.75 | F | 108 | 8/5/11 | 366 |
| A69.9002.13982 | 12.55 | F | 130 | 8/29/12 | 1090 |
| A69.9002.13984 | 23 | F | 141 | 8/29/12 | 887 |
| A69.9002.13988 | 73.22 | F | 131 | 8/29/12 | 1095 |
| A69.9002.13994 | 117.39 | M | 103 | 8/29/12 | 1078 |
| A69.9002.14000 | 73.82 | F | 136 | 8/29/12 | 1097 |
| A69.9002.13990 | 41.39 | F | 128 | 8/30/12 | 1089 |
| A69.9002.13992 | 70.73 | M | 119 | 8/30/12 | 1097 |
| A69.9002.13998 | 17.67 | M | 131 | 8/30/12 | 985 |
| A69.1303.33561 | 60.56 | -- | -- | -- | 831 |
| A69.1303.33562 | 26.32 | -- | -- | -- | 508 |
| A69.9001.30710 | 20.38 | -- | -- | -- | 1334 |

* All individuals had >100 detections and >10 month of data.

† -- indicates unrecorded data.

**Supplementary Figure 1.** Fishing effort (days, grey bars, left vertical axis) and sharks captured (individuals, red line, right vertical axis) at each of the 12 sampling occasions between 2006-2014.

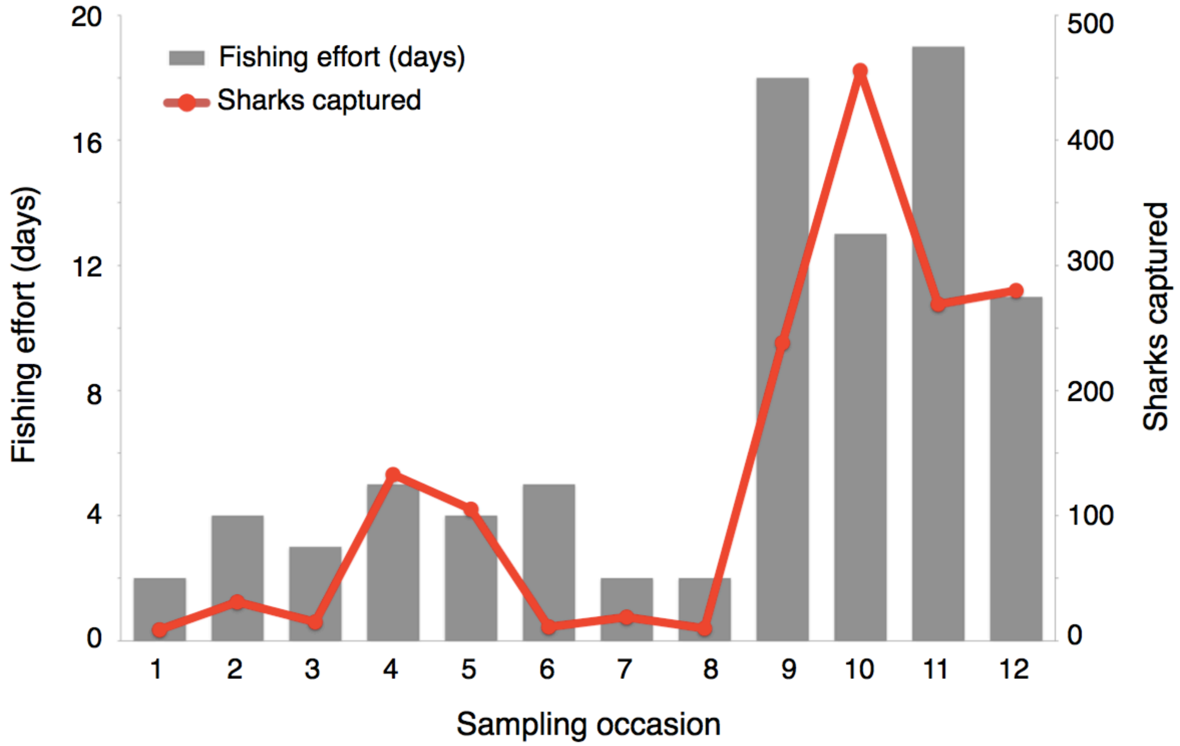

**Supplementary Figure 2.** Passive acoustic telemetry was used to monitor the movement of *C. amblyrhynchos* via an array of 76 individual underwater acoustic receivers (red triangles, VR2W, Vemco) that cover all of Palmyra's unique forereef, backreef, and lagoon habitats. Figure was created using R (version 3.1.3 [www.r-project.org/]). Habitat information is from the National Oceanic and Atmospheric Administration's (NOAA) National Centers for Coastal and Ocean Science (NCCOS) data collections 2016 (products.coastalscience.noaa.gov).

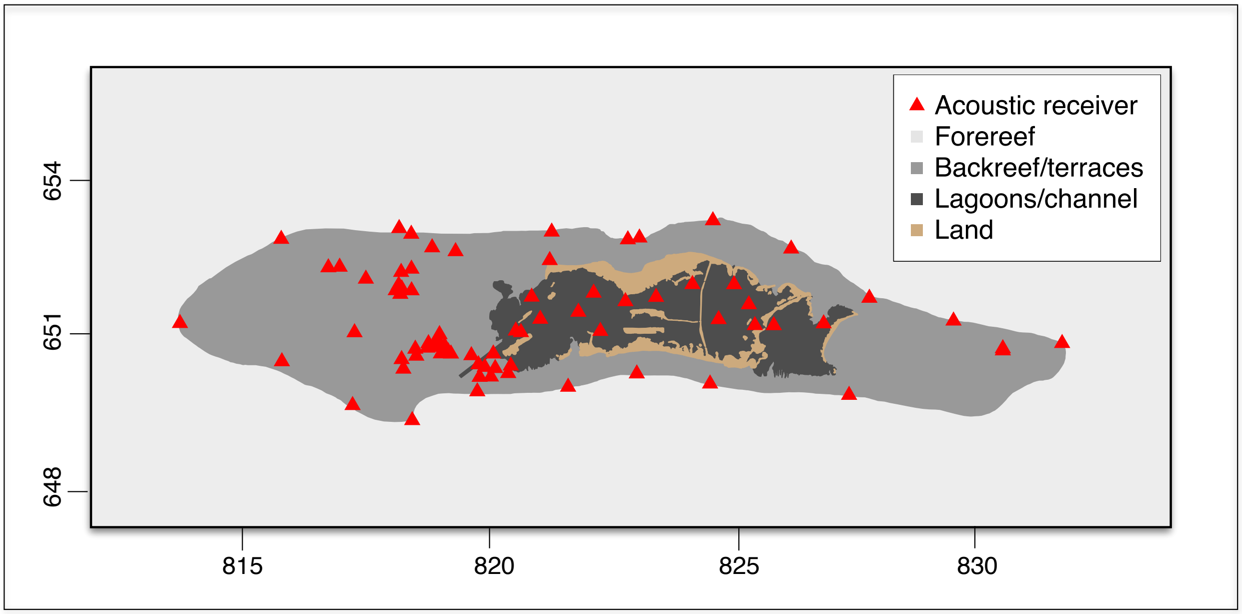

**Supplementary Figure 3.** Capture-recapture data from the western forereef and backreef (area contained within the yellow box) was used to estimate *C. amblyrhynchos* abundance and density through time. 774 individual sharks were captured in the region (578 female, 195 male, 1 unrecorded). Figure was created using R (version 3.1.3 [www.r-project.org/]). Habitat information is from NOAA's NCCOS data collections 2016 (products.coastalscience.noaa.gov).

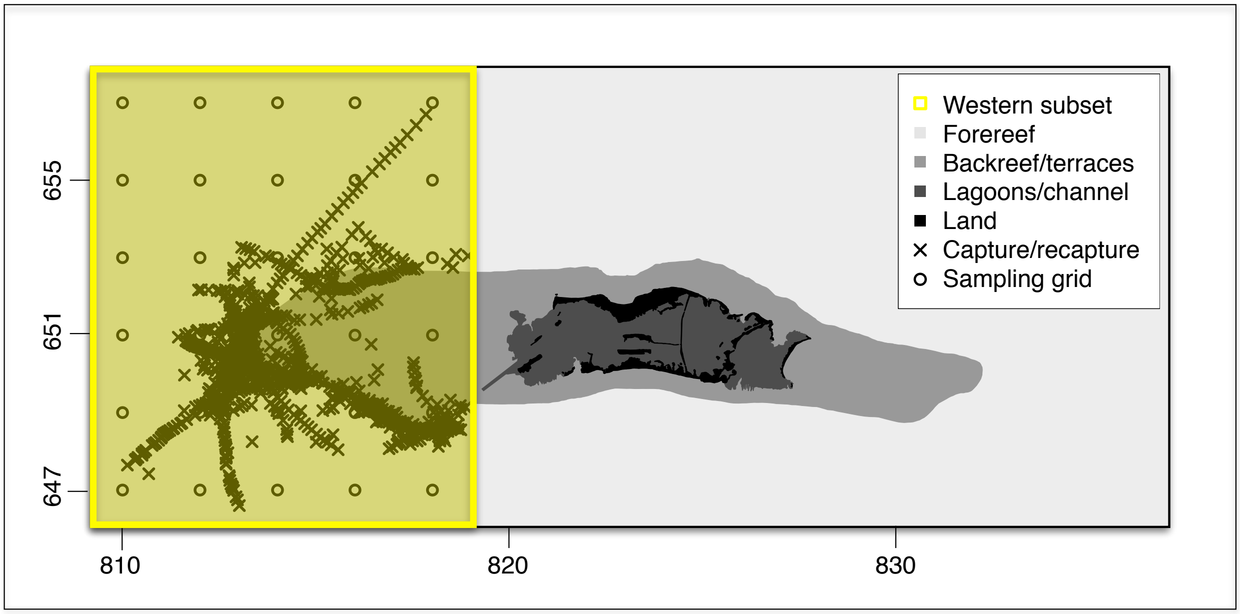

Supplement: Supplementary Information [file srep43131-s1.pdf]
